# A study of inhibitors of d-*glycero*-β-d-*manno*-heptose-1-phosphate adenylyltransferase from *Burkholderia pseudomallei* as a potential antibiotic target

**DOI:** 10.1080/14756366.2021.1900166

**Published:** 2021-03-18

**Authors:** Suwon Kim, Seri Jo, Mi-Sun Kim, Dong Hae Shin

**Affiliations:** College of Pharmacy and Graduate School of Pharmaceutical Sciences, Ewha W. University, Seoul, Republic of Korea

**Keywords:** d-*Glycero*-β-d-*manno*-heptose-1-phosphate adenylyltransferase, *Burkholderia pseudomallei*, ADP‐l‐*glycero*‐β‐d‐*manno*‐heptose, ChemBridge compounds, anti-melioidosis agent

## Abstract

d-*Glycero*-β-d-*manno*-heptose-1-phosphate adenylyltransferase from *Burkholderia pseudomallei* (*Bp*HldC) is the fourth enzyme in the ADP‐l‐*glycero*‐β‐d‐*manno*‐heptose biosynthesis pathway producing a lipopolysaccharide core. Therefore, *Bp*HldC is an anti-melioidosis target. Three ChemBridge compounds purchased from ChemBridge Corporation (San Diego, CA) were found to have an effective inhibitory activity on *Bp*HldC. Interestingly, ChemBridge 7929959 was the most effective compound due to the presence of the terminal benzyl group. The enzyme kinetic study revealed that most of them show mixed type inhibitory modes against ATP and βG1P. The induced-fit docking indicated that the medium affinity of ChemBridge 7929959 is originated from its benzyl group occupying the substrate-binding pocket of *Bp*HldC. The inhibitory role of terminal aromatic groups was proven with ChemBridge 7570508. Combined with the previous study, ChemBridge 7929959 is found to work as a dual inhibitor against both HldC and HddC. Therefore, three ChemBridge compounds can be developed as a potent anti-melioidosis agent with a novel inhibitory concept.

## Introduction

*Burkholderia* is a genus of Gram-negative bacteria, which has ecological, metabolic, and morphological diversity^1–4^. The *Burkholderia* genus belongs to the family *β-proteobacteria*, and *Burkholderia* has two major species, the Clade I and the Clade II. The most clinically important in these groups are the *B. cepacia* complex (BCC) and the *B. pseudomallei* group in the Clade I^4^. The BCC are pathogens that are opportunistic to immunodeficient patients and infection from those bacteria can be prevalent and fatal to cystic fibrosis patients^5–7^. Meanwhile, the *B. pseudomallei* group composes of four species, which are closely related (*B. pseudomallei*, *B. mallei*, *B. thailandensis*, and *B. oklahomensis*). The incidence of melioidosis has been reported in Thailand and northern Australia. In Thailand, it is in the high ranks among infectious diseases that cause the death of patients who had HIV/AIDS and tuberculosis. In northern Australia, it is the general reason for community-acquired pneumonia^8^.

Lipopolysaccharide (LPS) is an amphipathic glycolipid in the outer membrane of Gram-negative bacteria. Through electrostatic interactions with outer membrane proteins and divalent cations of phosphate groups on the heptoses, LPS maintains membrane stability and limits permeability of the bacterial outer membrane^9–11^. There are three parts composing the LPS layer: disaccharyl–lipid complex (lipid A); a core oligosaccharide; and a repeating oligosaccharide (O-antigen)^12^. The core oligosaccharide can be partitioned into an inner core consisting of 3-deoxy-d-*manno*-octulosonic acid (KDO) and heptoses, and an outer core containing hexoses and *N*-acetyl-d-hexosamine^13^.

The carbohydrate ADP‐l‐*glycero*‐β‐d‐*manno*‐heptose (ADP‐l‐β‐d‐heptose) is a substantial precursor of the inner core oligosaccharide part and is synthesised through the ADP‐l‐β‐d‐heptose biosynthesis pathway, which includes five enzymes^14^. d‐*Glycero*‐β‐d‐*manno*‐heptose‐1‐phosphate adenylyltransferase (HldC) is the fourth enzyme in the biosynthesis pathway. In many bacterial species, the enzymatic function of HldC is achieved by a bifunctional enzyme HldE composed of ATP-dependent kinase and adenylyltransferase domains. The former acts in step 2 and the latter in step 4 of the biosynthesis pathway^13^. However, in *B. pseudomallei*, *Neisseria meningitidis*, *N. gonorrhoeae*, *etc*., the two domains of the HldE protein are encoded by separate genes, *hldA* (kinase) and *hldC* (adenylyltransferase)^15^. Since the blockage of the HldC catalytic activity prevents the biosynthesis of heptoses of the inner core oligosaccharide of LPS, it can be a potential antibiotic target^13^. Thus, we screened chemical compounds for inhibitors and tried to deduce their essential structural properties to bind with inhibitors.

## Materials and methods

### Preparation of protein native *Bp*HldC

The gene of *Bp*HldC, *BphldC* (NCBI reference sequence: WP_004189202.1), coding for the *Bp*HldC protein was amplified by using primers and ligated into the amplified expression vector pB_2_ by way of ligation-independent cloning (LIC) method. To overexpress the protein, we transformed pDNAs into *Escherichia coli* (*E. coli*) BL21 (DE3). Transformed *E. coli* cells were incubated on Luria–Bertani (LB) agar plates. Several colonies were chosen and grown in test tubes with a cap to determine the condition for culturing in bulk. In the process, a cell stock was prepared and frozen. The mass culture proceeded at 310 K with shaking. As the absorbance at 600 nm of broth reached 0.6–0.8, isopropyl-β-d-1-thiogalactopyranoside was added for expression of *Bp*HldC. The cultures of *Bp*HldC were incubated at 298 K for 16 h in a shaking incubator. The expressed proteins contained non-cleavage N-terminal His_6_-tags followed by five glycines in *Bp*HldC (MHHHHHH GGGGG). To collect cells, we centrifuged the culture fluid with a high-speed refrigerated centrifuge at 7650×*g* (6500 rev min^−1^) for 10 min at 277 K. The cultured cell pellet was suspended and fragmentised using a Digital Sonifier 450 (Branson Ultrasonics Co., Danbury, CT). Cell debris was pelleted by centrifugation. Using a HisTrap column (GE Healthcare, Piscataway, NJ), affinity chromatography was done with the supernatant on an ÄKTA explorer system (GE Healthcare, Piscataway, NJ). Ion-exchange chromatography has been done as the secondary purifications using a 5 ml Hi-Trap Q column (GE Healthcare, Piscataway, NJ). The purified native proteins were concentrated to adequate concentration for use in the assay.

### Preparation of protein native HddC and KdsB

HddC proteins from *B. pseudomallei*, *Campylobacter jejuni*, *E. coli*, and *Prevotella sp. 10(H)* have been purified according to the previous method^16^. In brief, affinity chromatography was done and ion-exchange chromatography has followed as the secondary purifications. KdsB proteins from *B. pseudomallei*, *C. jejuni*, *Chlamydia psittaci*, *E. coli*, *Klebsiella aerogenes*, *N. meningitidis*, *Pseudomonas aeruginosa*, *Salmonella enterica*, and *Vibrio cholerae* have been purified according to the previous method^17^. Purification was also carried out with the same process. Glucose-1-phosphate thymidylyltransferase from *P. aeruginosa* has been also purified with a similar protocol as above sugar nucleotidyltransferases (SNTs).

### Chemical screening with a malachite green assay method

The screening of about 150 chemical compounds (Table S1) was performed with a malachite green assay method^18^. The principle of this method is: when SNTs transfer the AMP moiety from ATP to the heptose, ADP-d-*glycero*-β-d-*manno*-heptose and pyrophosphate (PPi) are produced. PPi was decayed into two phosphates by inorganic pyrophosphatase (IPP) and phosphates were measured by the malachite green method. β-d-Glucose-1-phosphate (βG1P) was purchased from Tokyo Chemical Industry Co. (Tokyo, Japan); TCI was used as a substrate because it is difficult to obtain the actual substrate, d-*glycero*-β-d-*manno*-heptose-1-phosphate (βH1P). The content of the α-form of this product was less than 0.1% in the current lot. A colour reagent of the malachite green method for phosphate detection was a mixture of ammonium molybdate ((NH_4_)_6_Mo_7_O_24_), malachite green solution and Tween 20 in the ratio 1:3:0.1. The mixture was filtered with a PVDF syringe filter and stood at room temperature (RT) for 1 h before use. All chemicals (25 μM) were tested for their inhibitory potential through a comparison of actual absorbances with control at 620 nm. The actual absorbance was obtained from the difference in absorbance between the reaction mixtures with and without *Bp*HldC. The reaction mixture included 10 mM Tris–HCl (pH 7.5), 10 mM MgCl_2_, 0.04 unit IPP, and 0.025 mg ml^−1^ *Bp*HldC (1.3 µM). To evaluate the accuracy of the inhibitor screening, *Z*′ factor was determined to be 0.9 (*n* = 15). The results indicate that the accuracy of the enzyme inhibitor test using this method is excellent^19^.

### Enzyme kinetics of *Bp*HldC

The malachite green assay method^18^ was used to study the steady-state kinetics of *Bp*HldC. For the kinetic studies, reaction mixtures including 5 mM Tris–HCl (pH 7.5), 10 mM MgCl_2_, 0.04 unit IPP, and 0.025 mg ml^−1^ *Bp*HldC with different concentrations of ATP (0.0039–1.5 mM) at the constant concentration of βG1P (1 mM) and with different concentrations of βG1P (0.0039–2.5 mM) at the constant concentration of ATP (0.5 mM) were used. After incubation at RT for an hour, 40 μl of reaction mixtures were mixed with the malachite reagent (160 μl). The mixtures were left for standing for 10 min to develop the colour. The standard curve fitting was performed using GraphPad Prism version 8.3.0 for Windows (GraphPad Software, San Diego, CA, www.graphpad.com)^20^.

### The IC_50_ values of ChemBridge compounds

The malachite green assay method was also used to study the dose-dependent inhibitory effect of ethyl 5-({[(5-benzyl-1,3,4-oxadiazol-2-yl)thio]acetyl}amino)-4-cyano-3-methyl-2-thiophenecarboxylate (ChemBridge 7929959), ethyl 5-[({[5–(4-chlorophenyl)-1,3,4-oxadiazol-2-yl]thio} acetyl)amino]-4-cyano-3-methyl-2-thiophene carboxylate (ChemBridge 7933420), and ethyl 4-cyano-5-[({[5–(2-ethoxyphenyl)-1,3,4-oxadiazol-2-yl]thio}acetyl)amino]-3-methyl-2-thiophenecarboxylate (ChemBridge 7991890) purchased from ChemBridge Corporation (San Diego, CA). The 40 μl of reaction mixtures containing 5 mM Tris–HCl (pH 7.5), 10 mM MgCl_2_, and 0.05 mg ml^−1^ *Bp*HldC with different concentrations of ChemBridge 7929959 (0.0245–100 μM), 7991890 (0.0245–250 μM), 7933420 (0.0245–250 μM) were incubated at RT for an hour. The reaction mixtures lacking *Bp*HldC with the same concentration of ChemBridge compounds (7929959, 7933420, and 7991890) used as above were also incubated at RT for an hour and measured as blanks. The reaction was initiated by adding 1 mM βG1P and 0.5 mM ATP and stood for an hour. After incubation, 160 μl of the same malachite reagent used above was added and left for standing for 10 min. The absorbance was measured at 620 nm using the microplate spectrophotometer (Spectramax 190, Molecular Devices Corporation, Sunnyvale, CA). The percentage reactivity (% Reactivity) was calculated depending on the difference in absorbance of whether *Bp*HldC was present or not in the reaction mixtures with ChemBridge compounds. The IC_50_ values of the ChemBridge compounds of *Bp*HldC were calculated and plotted by a nonlinear regression analysis using GraphPad Prism 8.3.0 (GraphPad Software, San Diego, CA).

### Inhibitory enzyme kinetics

ATP concentrations were varied at the range of 0.0078–1.5 mM and 1 mM βG1P, the saturated concentration, was mixed with the reaction mixture with 10 mM Tris buffer (pH 7.5) at RT in a 96-well microplate. On the other hand, when βG1P concentrations were varied at 0.0078–2.5 mM, the ATP concentration was constant at 0.5 mM. Components of the reaction mixture were the same as those of enzyme kinetic assay but compounds were also added with 0 µM (DMSO), 2.5 µM, 5 µM, 10 µM, 20 µM, and 40 µM, respectively. The data obtained from the spectrophotometer were used for fitting steady-state kinetic graphs and secondary plots using the GraphPad Prism program (GraphPad Software, San Diego, CA).

### Ligand preparation, target preparation, and induced-fit docking

All the docking and scoring calculations were executed using the Schrödinger software suite (Maestro, version 11.8.012). The ChemBridge compounds were extracted from the Hit2Lead homepage, ChemBridge Chemical Store, in SDF format and were combined in one file. The SDF files of βH1P and βG1P, substrates of *Bp*HldC, were got from the PubChem database. The files were imported into Maestro and prepared for docking using Ligand Preparation. The atomic coordinates of the crystal structure of *Bp*HldC (PDB ID: 5X9Q) were obtained from the Protein Data Bank and prepared by removing all solvent and adding hydrogens and minimal minimisation using Protein Preparation Wizard. Ioniser was used to generate an ionised state of all compounds at the target pH 7.0 ± 2.0. The input for an induced-fit docking is the prepared low-energy ligand forms. The induced-fit docking protocol^20^ was worked on the graphical user interface, Maestro 11.8.012 linked with the Schrödinger software. Receptor sampling and refinement were conducted for residues within 5.0 Å of each ligand for each ligand-protein complex. With Prime^21^, a side‐chain sampling and prediction module, as well as the backbone of the target protein, were energy minimised. A total of induced‐fit receptor conformations was generated for three ChemBridge compounds and two substates. Finally, the ligand poses were scored using a combination of Prime and Glide Score scoring functions^22^.

## Results

### Chemical screening with a malachite green assay method

**Figure 1.**
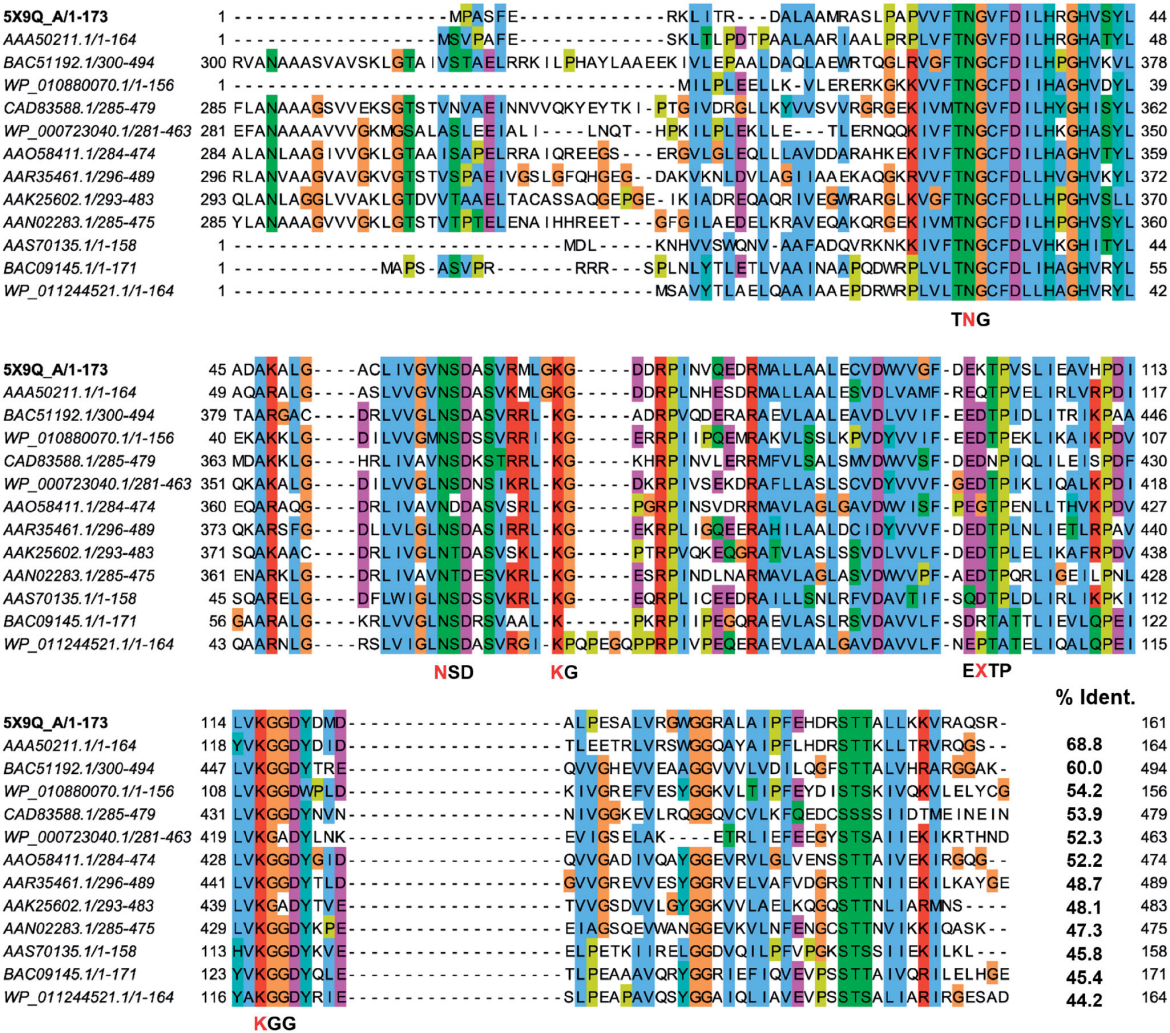
A multiple sequence alignment among *Bp*HldC and its homologs. The predicted d-*glycero*-β-d-*manno*-heptose-1-phosphate (βH1P) binding sites were based on the docking experiment obtained after selecting 12 *Bp*HldC homologs. The predicted substrate-binding motifs, TNG, NSD, KG, EXTP, and KGG, are indicated and the red characters represent the residues that took part in the interaction with βH1P. In the case of the EXTP motif, X represents any amino acids. In the docking result, the X residue is Lys100. Interestingly, the backbone oxygen was involved in the interaction with βH1P.

**Figure 2.**
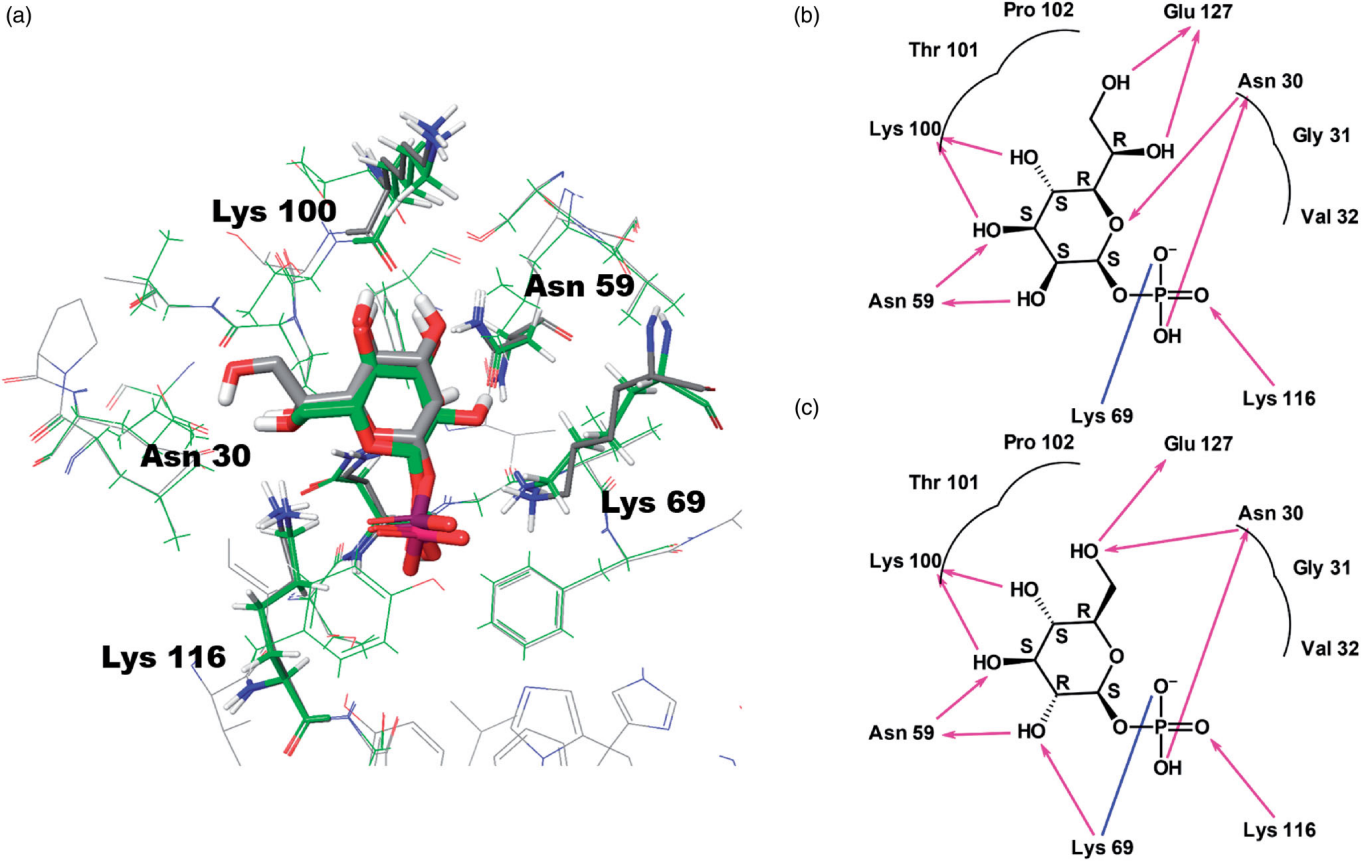
Predicted docking modes of d-*glycero*-β-d-*manno*-heptose-1-phosphate (βH1P) and βG1P in the catalytic site of *Bp*HldC. The presumed substrate binding sites were obtained based on the X-ray crystal structure of three homologues complexed with their substrates (PDB ID: 1H1T, 1N1D, 3HL4). (a) The superimposed view of βH1P and βG1P docked on the active site (grey, βH1P; green, βG1P). The substrates and functionally important residues Asn 30, Asn 59, Lyn 69, Lys100, and Lys 116 were depicted with a stick model. All residues belong to putative substrate-binding motifs (Figure 1). In the case of Lys100, the backbone oxygen participates in the interaction. In addition, 2D schematic representations of the docked (b) βH1P and (c) βG1P with *Bp*HldC were drawn. Figures were created with Maestro v11.5.011 and ChemDoodle v10.1.0 (iChemLabs^TM^). The blue line represents ion interactions and the pink arrows represent the hydrogen bonds. According to the induced-fit docking results, the glide *g*-scores of substrates in the binding site pocket were –6.68 for βH1P and –6.59 for βG1P.

The enzyme kinetic study of *Bp*HldC was performed and the chemical screening of about 150 compounds was done with the malachite green assay method. In this assay, the amount of inorganic phosphate was measured after pyrophosphatase treatment. It is a fast, reproducible, colorimetric method for measuring inorganic free phosphate in aqueous solutions. Hence, the assay offered great ease of exploring potent inhibitors against *Bp*HldC. A list of compounds is provided in Table S1 of the Supporting Information. In order to perform the assay, a natural substrate was required. Unfortunately, the natural substrate of HldC, βH1P, was not commercially available. Even worse, the closest derivative of the natural substrate, β-d-mannose-1-phosphate (βM1P), also was not commercially available. Nevertheless, the problem could be overcome by using βG1P as an alternative substrate. The feasibility of βG1P as a surrogate of βH1P was tested with an *in silico* docking experiment. The putative substrate-binding pocket was predicted using approximately 5000 homologue sequences of *Bp*HldC with more than ∼44% sequence identity (Figure 1). Three substrate-complexed structures (PDB ID: 1N1D, 1H1T, 3HL4) of *Bp*HldC homologues were also considered since their high overall topological similarity as shown in DALI *z*-scores; 16.0, 10.7, and 12.9, respectively. Two compounds display similar binding modes at the catalytic pocket as shown in Figure 2. Luckily, βG1P worked as an alternative to βH1P for HldC. Therefore, βG1P was provided as a substrate to assay HldC. A similar approach was reported for searching inhibitory compounds of d-*glycero*-α-d-*manno*-heptose-1-phosphate guanylyltransferase from *Yersinia pseudotuberculosis* (*Yp*HddC)^16^. α-d-Mannose-1-phosphate (αM1P) behaved effectively as a surrogate substrate instead of the natural substrate of *Yp*HddC, d-*glycero*-α-d-*manno*-heptose-1-phosphate. After a round of screening, three ChemBridge compounds (7929959, 7933420, and 7991890) blocking the catalytic activity of *Bp*HldC were detected as potential inhibitors.

### Enzyme kinetics of *Bp*HldC

**Figure 3.**
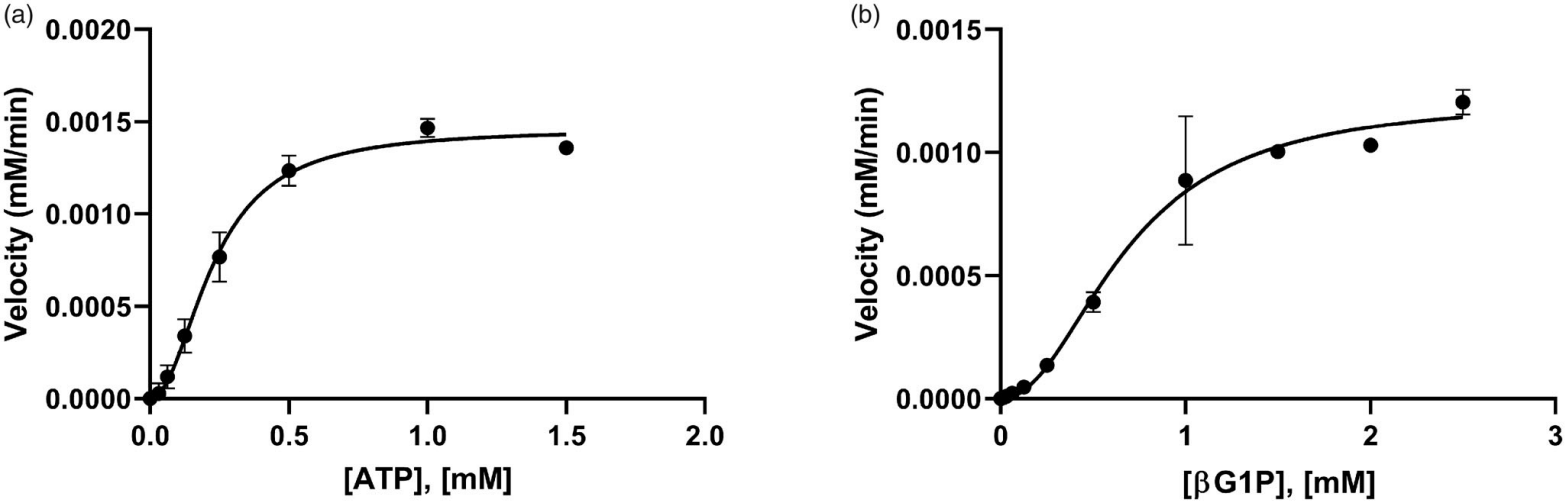
The allosteric activities of *Bp*HldC. The enzyme activities with (a) various concentrations of ATP with 1 mM βG1P and (b) various concentrations of βG1P in the presence of 0.5 mM ATP. The graphs indicated that the maximum velocities were obtained at 0.5 mM ATP in the presence of 1 mM βG1P and 1 mM βG1P in the presence of 0.5 mM ATP, respectively. Each dot is expressed as the mean ± standard error of the mean (*n* = 3).

To determine the kinetic parameters of *Bp*HldC for βG1P and ATP, enzyme assays were performed with various concentrations of ATP at a constant βG1P concentration (1 mM) and with various concentrations of βG1P at a constant ATP concentration (0.5 mM). The graphs of the velocities vs. concentrations of βG1P and ATP are shown in Figure 3. Both substrates vs. velocity plots of *Bp*HldC were well matched with an allosteric sigmoidal model. The kinetic parameter values of *Bp*HldC with respect to ATP at the steady-state under the constant concentration of βG1P (1 mM) were 0.23 ± 0.02 mM for *K*_half_ (equals to EC_50_) and 0.05 ± 0.02 mM for *K*_prime_ (related to the *K*_m_). Those of *Bp*HldC with respect to βG1P under the constant concentration of ATP (0.5 mM) were 0.68 ± 0.07 mM for *K*_half_ and 0.45 ± 0.14 mM for *K*_prime_. All parameters were calculated using the GraphPad Prism software (San Diego, CA).

### The IC_50_ values of ChemBridge compounds

**Figure 4.**
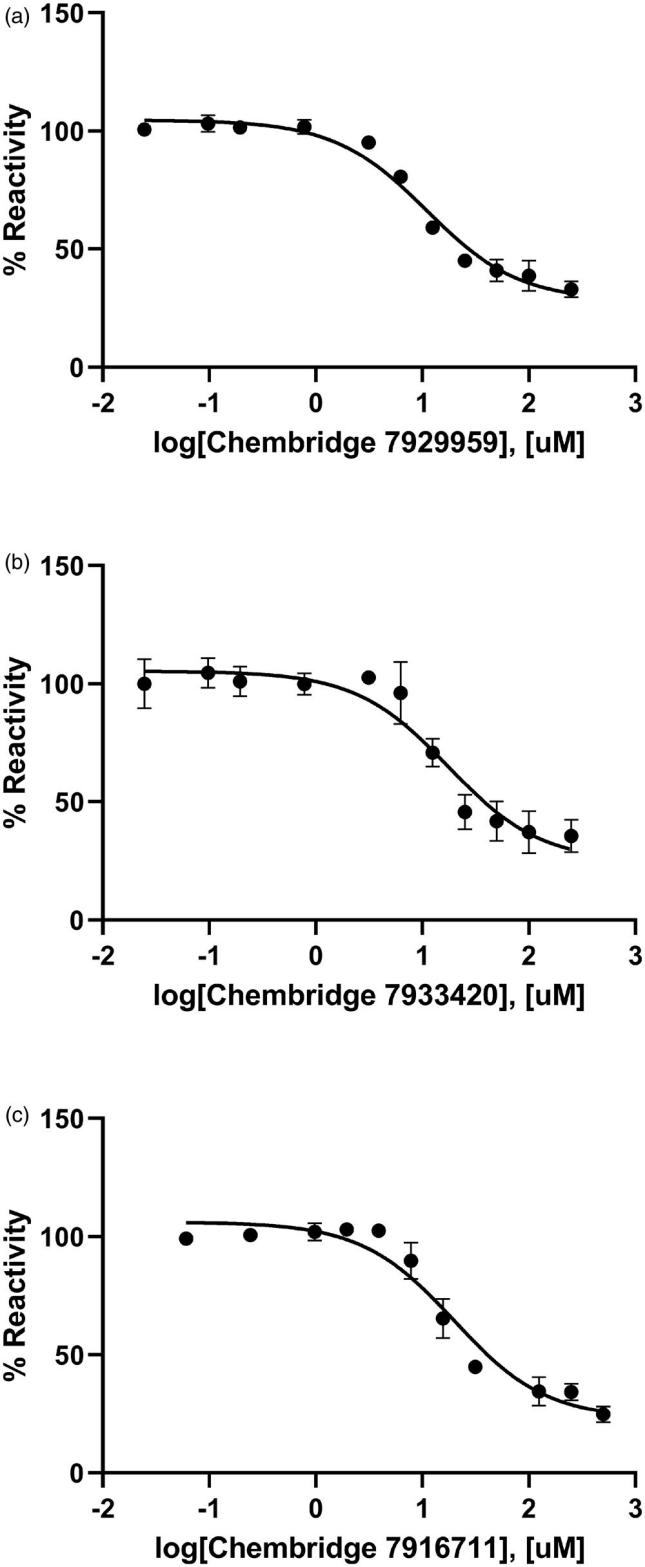
Dose-dependent inhibitory curves for the inhibition of *Bp*HldC by ChemBridge (a) 7929959, (b) 7933420, and (c) 7991890. Each data point represents the effect of each inhibitory compound against *Bp*HldC compared to the control. The %Reactivities are plotted against the log-concentration of inhibitory compounds. Each dot is expressed as the mean ± standard error of the mean (*n* = 3).

The inhibitory effect of three ChemBridge compounds against *Bp*HldC was found through the chemical screening with the malachite green assay method. Therefore, the dose-dependent inhibitory effect of the ChemBridge compounds was pursued and estimated at the saturated substrate concentrations (0.5 mM ATP and 1 mM βG1P). The results were plotted as log inhibitor concentration vs. percentage reactivity calculated from absorbance (Figure 4). IC_50_ values determined from the dose-dependent inhibitory curves of ChemBridge compounds against *Bp*HldC: ChemBridge 7929959 was 10.92 µM; ChemBridge 7933420 was 17.46 µM; ChemBridge 7991890 was 47.11 µM.

### Inhibitory enzyme kinetics

**Figure 5.**
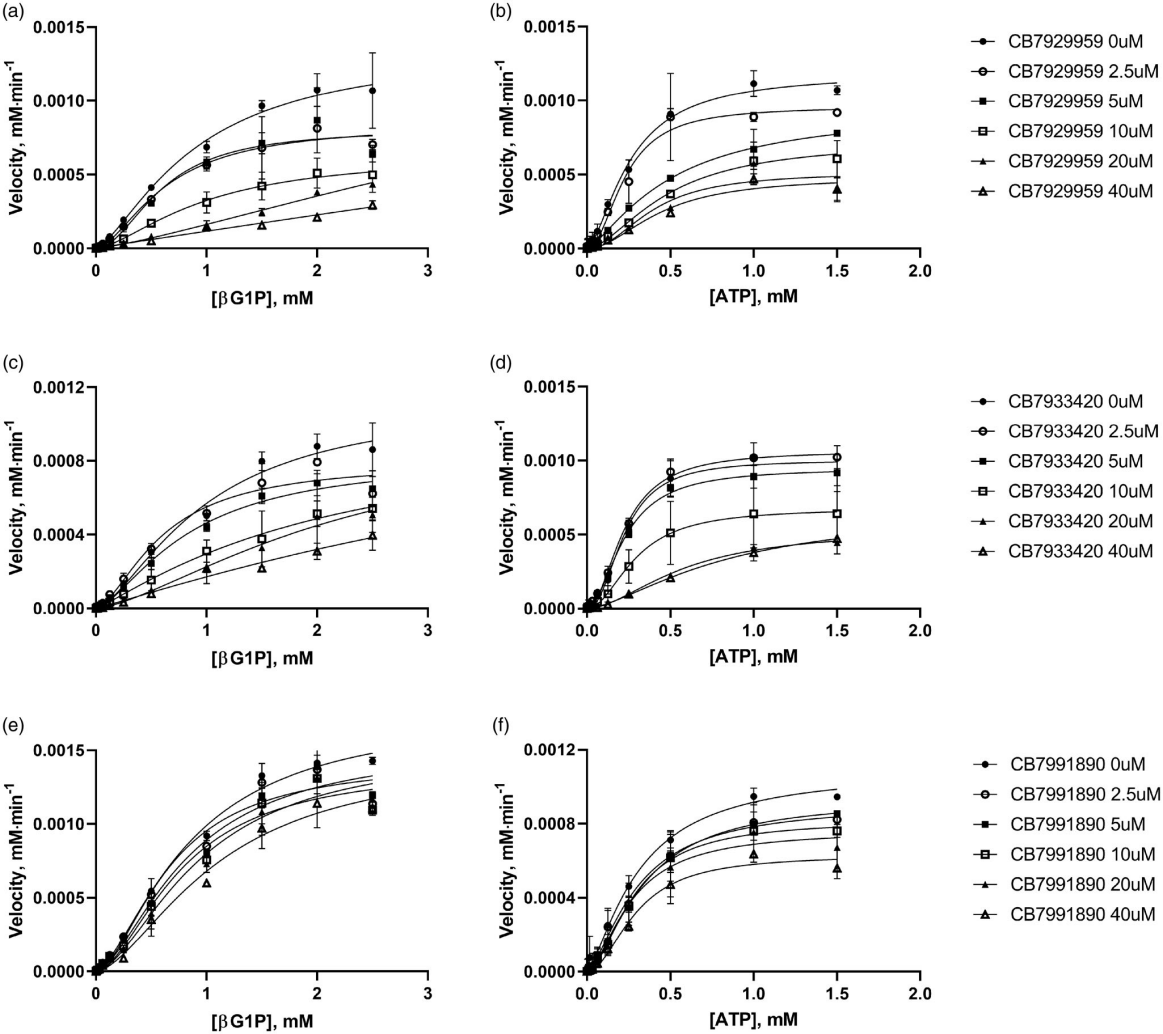
Inhibitory enzyme kinetics of *Bp*HldC with ChemBridge 7929959 (CB7929959) (a, b), ChemBridge 7933420 (CB7933420) (c, d), and ChemBridge 7991890 (CB7991890) (e, f). (a, c, e) Steady-state kinetics with inhibitors regarding ATP at a fixed concentration of 1 mM βG1P. (b, d, f) Inhibition of *Bp*HldC by compounds at varying βG1P concentrations with a constant concentration of 0.5 mM ATP is presented. Compounds were treated to each point with six concentrations (0, 2.5, 5, 10, 20, and 40 µM). Each dot is expressed as the mean ± standard error of the mean (*n* = 3).

The inhibitory kinetic assay was carried out with varying concentrations of one substrate (ATP) at the fixed concentration of the other substrate (βG1P) in different concentrations or vice versa regarding three ChemBridge compounds. Raw data were obtained using the spectrophotometer. Steady-state inhibitory enzyme kinetics plots were fitted by the allosteric sigmoidal model (Figure 5).

The secondary plots were used to determine the inhibitory profile of *Bp*HldC^23^. Alpha values (the ratio of *K_i_′*/*K_i_*) classifying inhibitory modes of inhibitor compounds were obtained with inhibition constants (*K_i_′* and *K_i_*) determined from 1/*V*_max_ vs. [*I*] for *K_i_′* and slope (*K*_m_/*V*_max_ ≈ *K*_prime_/*V*_max_) vs. [*I*] for *K_i_* based on the graphs from Figure 5. Inhibitors with an approximate alpha value of 1.0 belong to a non-competitive type. The values which get to zero and which get to infinite statistically correspond to uncompetitive and competitive types, respectively. The mixed type model is determined when the alpha value exceeds 1.0. The results for ChemBridge compounds on *Bp*HldC are shown in Table 1. The inhibition type of ChemBridge compounds appeared in the mixed-type or uncompetitive type inhibition mechanisms for ATP and the mixed-type inhibition for βG1P.

**Table 1.** Inhibitory properties of compounds with *Bp*HldC^a^.

Compounds	ATP			βG1P		
	*K_i_′* (µM)	*K_i_* (µM)	Type	*K_i_′* (µM)	*K_i_* (µM)	Type
ChemBridge 7929959	15.59	0.43	Mixed type^b^	11.08	0.15	Mixed type
ChemBridge 7933420	18.46	6.80	Mixed type	568.8	6.02	Mixed type
ChemBridge 7991890	56.46	Infinite^c^	U.C.^d^	50.81	39.55	Mixed type

^a^ All parameters were calculated using GraphPad Prism program (GraphPad Software, San Diego, CA).

^b^ The mixed type includes properties of both competitive and non-competitive inhibition^23^.

^c^ The graph was close to parallel to the *X*-axis.

^d^ U.C.: un-competitive type.

### Ligand preparation, target preparation, and induced-fit docking

**Figure 6.**
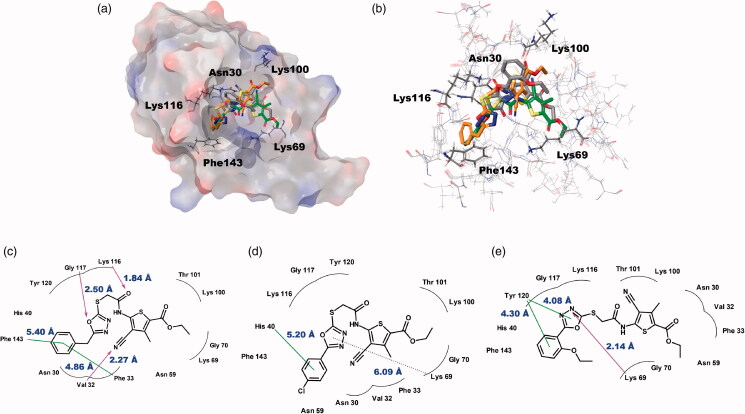
Predicted docking modes of ChemBridge compounds in the catalytic site of *Bp*HldC. (a) The docking pose of ChemBridge 7929959 (orange), 7933420 (green), and 7991890 (grey) were depicted on the electrostatic surface potential of *Bp*HldC (red, negative; blue, positive; white, uncharged). (b) The superimposed view of ChemBridge compounds docked on the active site. Functionally important residues Asn 30, Lys69, Lys100, Lyn116, and Phe143 were depicted with a stick model according the docking mode of ChemBridge 7929959. In addition, 2D schematic representations of the docked ChemBridge compounds with *Bp*HldC were drawn. (c) ChemBridge 7929959, (d) ChemBridge 7933420, and (e) ChemBridge 7991890. Figures were created with Maestro v11.5.011 and ChemDoodle v10.1.0 (iChemLabs^TM^). The pink arrows represent the hydrogen bonds. The green line represents the π–π interaction and the dotted line represents the π–cation interaction. Comparison with other substrate-bound crystal structures of *Bp*HldC homologues revealed that Phe143 former locates in the nucleotide binding cavity and Lys69 interacts with the phosphate moiety of nucleotide. The residues in the highly conserved substrate binding motifs (Figure 1) are drawn with curved lines.

To deduce the binding modes of ChemBridge compounds with *Bp*HldC at a molecular level, an in-depth theoretical investigation with an induced-fit docking study using the Schrödinger program was carried out (Figure 6(a,b)). The crystal structure of *Bp*HldC deposited in the Protein Data Bank (PDB ID: 5X9Q) was retrieved and docked with ChemBridge compounds to predict their binding modes. In the asymmetric unit of the crystal structure of *Bp*HldC, there are four chains and two of them were determined with 4-morpholineethanesulfonic acid (MES) of which the sulphonic acid moiety bound with Lys69 at the centre of the active site composed of sugar and nucleotide-binding pockets. We used the chain D for docking because it contains MES and mimics a substrate-bound closed conformation. The docking pocket was defined based on the structural comparison of *Bp*HldC with its homologues complexed with their substrates (PDB ID: 1N1D, 1H1T, 3HL4). As a result, putative substrate and nucleotide-binding cavities of *Bp*HldC were searched. Top-ranked structures with the highest glide *g*-scores from the induced-fit docking results were surveyed. According to the induced-fit docking results, the glide *g*-scores of structures that were reasonably docked in the active site pocket were −8.30 for ChemBridge 7929959, −7.38 for ChemBridge 7933420, and −5.23 for ChemBridge 7991890. The predicted complex structures and 2D schematic representations are illustrated in Figure 6(c–e). The docking study with the three ChemBridge compounds indicated that the order of the glide *g*-scores was well-matched with their experimental inhibitory capability (IC_50_).

## Discussion

The ADP‐l‐β‐d‐heptose biosynthesis pathway is one of three major pathways including GDP-6-deoxy-α-d-*manno*-heptose and CMP-3-deoxy-d-*manno*-octulosonic acid biosynthesis pathways, which produces carbohydrates in the core oligosaccharide part of LPS. We assayed inhibitory activities of compounds with an in-house chemical library (Table S1) based on the expanded version of the previous one^16^ against the adenylyltransferase activity of HldC from *B. pseudomallei* (*Bp*HldC). We found that compounds having a similar scaffold showed inhibitory activities against *Bp*HldC. Hence, we attempted to bring out the structural meaning and move towards the development of inhibitors that have a better affinity.

In brief, the docking modes of three chemicals can explain their inhibitory properties (Table 1). At first, the benzyl moiety of ChemBridge 7929959 and the phenyl moiety of 7933420 do not occupy the whole nucleotide-binding pocket. Instead, they occupy where the ribose moiety of ATP locates. Therefore, they do no work as a competitive inhibitor regarding ATP but as a mixed type. In the case of ChemBridge 7991890, the phenyl moiety is protruded outward due to the ethoxy group. As a result, the nucleotide-binding pocket is more spacious than the above two compounds. Therefore, the compound seems to function as an uncompetitive inhibitor interacting with a catalytic product. Second, the substrate-binding pocket is not directly blocked by three compounds. The curved backbone of compounds occupies the interface of the substrate-binding pocket. Therefore, their inhibitory mode can be explained by a mixed type. It is worth to note that epigallocatechin gallate (EGCG) displays a competitive binding mode against both β-glucose-1-phosphate and ATP by occupying some parts of the substrate and nucleotide-binding regions^24^. Therefore, these *in silico* docking data provide a rational background to explain different binding modes of ChemBridge compounds and flavonoids.

The small *K_i_* value of ChemBridge 7929959 indicates that it binds well in the substrate-binding site. Val32, Lys116, and Gly117 interact strongly with the compound compared with the other two compounds. In addition, Phe143 on the FEHDRSTT motif was predicted to have a critical role to recruit ATP through π–π stacking interaction^14^. These properties seem to explain its 5.7- and 8.5-fold inhibitory activity compared with ChemBridge 7933420 and ChemBridge 7991890, respectively. The similar inhibitory mode of ChemBridge 7929959 and ChemBridge 7933420 may be originated from their benzyl and phenyl groups inserted into the hydrophobic pocket lined with Phe33, Tyr43, Leu44, Val56, and Leu85 (Figure 6). Actually, the morpholine moiety of MES is also located in the hydrophobic core^14^. However, the interaction of the oxadiazole ring of ChemBridge 7991890 with Lys69 and Tyr120 drew the 2-ethoxyphenyl group from the hydrophobic cavity. As a result, its position is a bit shifted to the surface compared with the other two ChemBridge compounds.

**Figure 7.**
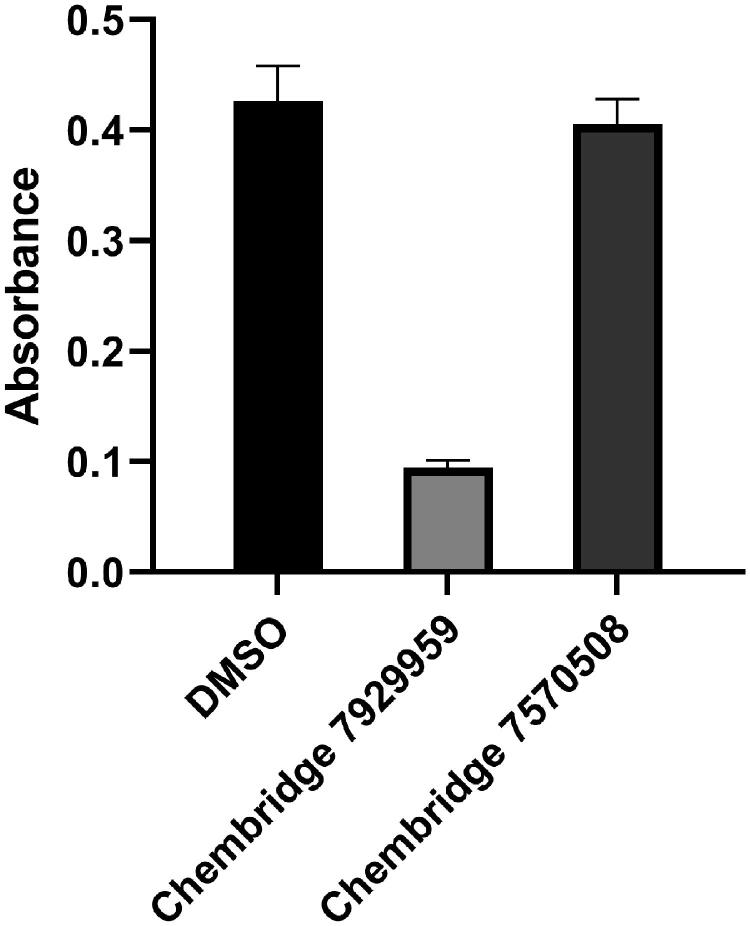
The comparison of reactivities of *Bp*HldC with ChemBridge 7929959 and 7570508. The malachite assay was performed with 0.5 mM ATP and 1 mM βG1P. The observed absorbance could be considered as the reactivity of *Bp*HldC. Compared to the control with DMSO, the reactivity of *Bp*HldC with ChemBridge 7929959 fell meaningfully. However, the reactivity of *Bp*HldC with ChemBridge 7570508 was almost not changed.

The catalytic activity of *Bp*HldC was suppressed by ChemBridge 7929959 and its derivatives, ChemBridge 7933420 and 7991890. Therefore, the ChemBridge compounds possessed a molecular character strongly interacting with *Bp*HldC. A detailed structural comparison was performed to guess which portion of the ChemBridge compounds was essential to inhibit the nucleotidyltransferase activity of *Bp*HldC. The three ChemBridge compounds shared the core structure, ethyl 5-[({[1,3,4-oxadiazol-2-yl]thio}acetyl)amino] 4-cyano-3-methyl −2-thiophene carboxylate, except the terminal aromatic groups (Figure 6(c–e)). Unlike the terminal benzyl ring of ChemBridge 7929959, ChemBridge 7933420 contains a chlorophenyl ring and an ethoxy phenyl ring for ChemBridge 7991890. In order to identify whether the terminal aromatic group is crucial to inhibit the catalytic activity of *Bp*HldC or not, a commercially available compound with the closest core structure was purchased and assayed. It was ethyl 4-cyano-3-methyl-5-{[(4H-1,2,4-triazol-3-yl-thio)acetyl]amino}-2-thiophenecarboxylate (ChemBridge 7570508) with exactly the same chemical formula with the core structure of the three ChemBridge compounds except for triazole at the terminal end. Therefore, the polarity of the terminal end is converted from negatively polar to neutral in ChemBridge 7570508. Nevertheless, the binding capability of the common core structure could be predictable because the polarity change is ignorable considering the size of the compounds. Intriguingly, the inhibitory activity of ChemBridge 7570508 was almost absent in the assay (Figure 7). The result clearly indicates that the aromatic rings (the benzyl or phenyl groups) in the three ChemBridge compounds are essential for binding together with endowing their different binding affinity to *Bp*HldC (Figure 6).

**Figure 8.**
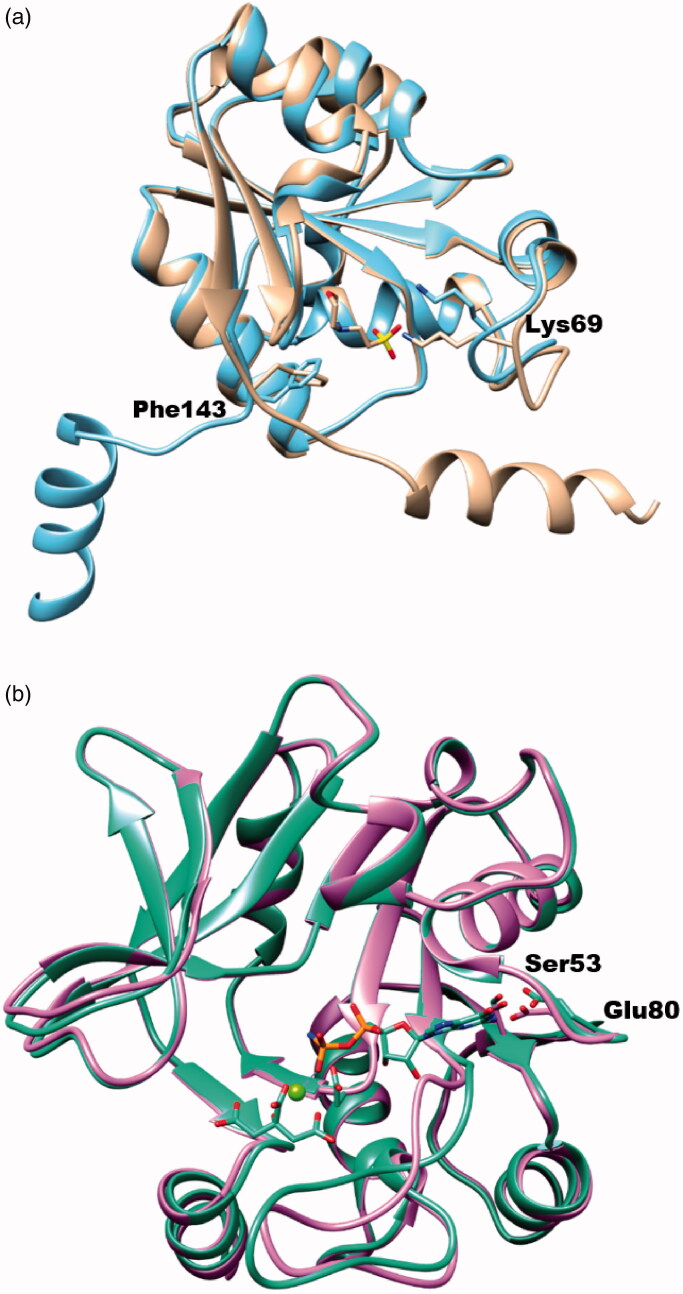
The superimposed ribbon diagrams of ligand-free and ligand-bound X-ray crystal structures of *Bp*HldC and *Yp*HddC. (a) The native (sky blue) and MES bound (tan) forms of *Bp*HldC were drawn. (b) The native (orchid) and GMPPN (spring green) bound forms of *Yp*HddC were drawn. A magnesium ion and a citrate molecules are also displayed. In both figures, catalytically important residues Lys69 and Phe143 were drawn with a stick model and labelled.

In the previous study, we figured out that ChemBridge 7929959 inhibits *Yp*HddC, the fourth enzyme of the GDP-6-deoxy-α-d-*manno*-heptose biosynthesis pathway^16^. However, their catalytic mechanisms are totally different. In the case of *Yp*HddC, the catalytic site is designed to accept GTP in a lock-and-key fashion. In contrast, *Bp*HldC adopts an induced-fit fashion triggering a huge conformational change of the C-terminal helix to perform its catalytic activity (Figure 8). As a result, their active site motifs and catalytic residues are different. Nevertheless, ChemBridge 7929959 functions as a dual inhibitor against *Bp*HldC and *Yp*HddC. The additional enzymatic studies have been performed to discern non-specific characteristics of the compound against various SNTs. However, ChemBridge 7929959 could not inhibit glucose-1-phosphate thymidylyltransferase from *P. aeruginosa*, HddCs from *B. pseudomallei*, *C. jejuni*, *E. coli*, and *P. sp. 10(H)* and KdsBs from *B. pseudomallei*, *C. jejuni*, *C. psittaci*, *E. coli*, *K. aerogenes*, *N. meningitidis*, *P. aeruginosa*, *S. enterica*, and *V. cholerae*. Therefore, the compound shows selectivity against *Bp*HldC and *Yp*HddC. Since the dual inhibitory property is beneficial as antibiotics, a further study is going on to develop antibiotics targeting both HldC and HddC from the same pathogenic species. Currently, derivatives removing the ethyl-2-thiophenecarboxylate moiety from the core structure are going on to define a minimal functional scaffold. In addition, derivatives adding functional groups to the benzyl moiety are being synthesised as a strategy to improve their binding affinity.

## Supplementary Material

Supplemental Material
