# Supplementary material for: A study of inhibitors of d-glycero-β-d-manno-heptose-1-phosphate adenylyltransferase from Burkholderia pseudomallei as a potential antibiotic target
Source: J Enzyme Inhib Med Chem. 2021 Mar 18;36(1):776–84. doi: 10.1080/14756366.2021.1900166 (PMC7993394; doi:10.1080/14756366.2021.1900166)
Supplement: Supplemental Material [file IENZ_A_1900166_SM9404.pdf]

Journal of Enzyme Inhibition and Medicinal Chemistry

A study of inhibitors of D-*glycero*-β-D-*manno*-heptose-1-phosphate adenylyltransferase from *Burkholderia pseudomallei* as a potential antibiotic target

Suwon Kim[a], Seri Jo[a], Mi-Sun Kim[a] and Dong Hae Shin[a]*

[a]*College of Pharmacy and Graduate School of Pharmaceutical Sciences, Ewha W. University, 52, Ewhayeodae-gil, Seodaemun-gu, Seoul 03760, Republic of Korea*

Corresponding author

Prof. Dong Hae Shin

Department of Pharmacy, Ewha W. University, Seoul, 03760, Republic of Korea

Tel: +82-2-3277-4502

Fax: +82-2-3277-2851

E-mail: dhshin55@ewha.ac.kr

Table S1. A home-made chemical library

No.	Name of compound
1	2',7'-Dichlorofluorescein Sodium
2	2-Pyridinealdoxime methochloride (2-PAM)
3	2,4-Dinitrophenylhydrazine
4	2,4,6-trimethyl-7-oxo-1,3,5-cycloheptatrien-1-yl 4-chlorobenzoate (Chembridge 5945310)
5	3,3',5,5'-Tetramethylbenzidine
6	4-(2-Hydroxyethyl)-1-piperazinepropanesulfonic acid (EPPS)
7	4-Vinylpyridine
8	5-Azacytidine crystalline
9	5-Nitroindole
10	6-bromochromone-3-carbonitrile
11	6-Diazo-5-oxo-L-norleucine
12	7-Isopropoxy-3-phenyl-4H-1-benzopyran-4-one (Ipriflavone)
13	8-Hydroxyquinoline
14	9-(4'-Dimethylaminophenyl)-2,6,7-trihydroxyfluorone sulfate hydrate
15	9-Aminoacridine hydrochloride
16	Acriflavine
17	Actinomycin D from *Streptomyces sp*.
18	Acyclovir
19	Adenosin 5'-(β-γ-imido) triphosphate (AMPPNP)
20	Amphotericin B
21	Ampicillin
22	Angelicin
23	Antipain
24	Aphidicolin from *Nigrospora sphaerica*
25	Aprotinin
26	Azaserine
27	Bacitracin zinc salt from *Bacillus licheniformis*
28	Borane dimethylamine complex
29	Calcium Ionophore A23187
30	Carbadox

31	Cefazolin
32	Cefoperazone
33	Cefotaxime
34	Chloramphenicol
35	Chloroquine
36	Chlorpromazine
37	Chlortetracycline
38	Cinoxacin
39	cis-5,8,11,14,17-Eicosapentaenic acid
40	Costunolide
41	Crystal Violet
42	Cycloheximide
43	Cytosine β-D-arabinofuranoside hydrochloride
44	D-(+) Galactosamine
45	D-Fructose 1,6-bisphosphate
46	D-Fructose 6-phosphate
47	D-Ribose 5-phosphate
48	Dehydrocostus lactone
49	Dimethylethylammoniumpropane sulfonate (NDSB-195)
50	Dimetridazole
51	Docosahexaenoic acid
52	Erythromycin
53	Ethyl 4-cyano-3-methyl-5-({[4- methyl-2- pyrimidinyl)thio]acetyl}-2-thiophenecarboxylate (ChemBridge 7698174)
54	Ethyl 4-cyano-5-[({[5-(2-ethoxyphenyl)-1,3,4-oxadiazol-2-yl]thio}acetyl)amino]-3-methyl-2-thiophenecarboxylate (ChemBridge 7991890)
55	Ethyl 4-cyano-3-methyl-5-{[(4H-1,2,4-triazol-3- ylthio)acetyl]amino}-2- thiophenecarboxylate (Chembridge 7570508)
56	Ethyl 5-({[(5-benzyl-1,3,4-oxadiazol-2-yl)thio]acetyl}amino)-4-cyano-3-methyl-2-thiophenecarboxylate (ChemBridge 7929959)
57	Ethyl 5-[({[5-(4-chlorophenyl)-1,3,4-oxadiazol-2-yl]thio}acetyl)amino]-4-cyano-3- methyl-2-thiophenecarboxylate (ChemBridge 7933420)
58	Ethyl acetate
59	Ethylene glycol
60	FCLA Free Acid (Chemiluminescence Reagent)
61	Fluorescein

62	Fusaric acid from *Gibberella fujikuroi*
63	G 418 disulfate
64	Gastrodin
65	Geniposide
66	Geniposidic acid
67	Gentamicin
68	Guanosine 5′-[β,γ- imido]triphosphate (=GMPPNP)
69	Hygromycin B from *Streptomyces hygroscopicus*
70	Ibuprofen
71	Indomethacin
72	Iodoacetic acid
73	Irgasan
74	Ivermectin
75	Josamycin
76	Kanamycin Sulfate
77	L-Ascorbic acid
78	L-Homocitrulline.
79	L-Lysine ethyl ester dihydrochloride
80	Levofloxacin
81	Lincomycin hydrochloride
82	Liriodendrin
83	Lysostaphin from *Staphylococcus staphylolyticus*
84	Metronidazole
85	Mevastatin
86	Mycophenolic acid
87	N-(6-Aminohexyl)-5-chloro-1-naphthalenesulfonamide Hydrochloride (W-7 Hydrochloride)
88	N-[2-(p-Bromocinnamylamino)ethyl]-5-isoquinolinesulfonamide dihydrochloride<br>(H-89 dihydrochloride)
89	N-Hydroxy-1,8-naphthalimide
90	n-Propyl Gallate
91	Nalidixic acid
92	Neohesperidin dihydrochalcone
93	Neomycin
94	Nile Blue A

95	Ochratoxin A from *Petromyces albertensis*
96	Orientin
97	Orotic acid
98	Oroxin B
99	Paclitaxel
100	Pectolinarin
101	Penicillin G
102	Phenazine methosulfate
103	Phenylbutazone
104	Phenylfluorone
105	Pinoresinol diglucoside
106	Polyvinylpyrrolidone
107	Poncirin
108	Praziquantel
109	Prinomastat
110	Prothionamide
111	Puerarin
112	Puromycin dihydrochloride from *Streptomyces alboniger*
113	Pyrantel pamoate
114	Pyrazinecarboxamide
115	Quercetin 3-β-D-glucoside
116	Quercetin hydrate
117	Quercitrin hydrate
118	Raltegravir
119	Rhodamine 6G
120	Rhoifolin (Apigenin 7-O-neohesperidoside)
121	Rifamycin SV sodium
122	Sodium deoxycholate
123	Sorbic acid
124	Spectinomycin
125	Streptomycin
126	Streptozocin
127	Sulfachloropyridazine

128	Sulfathiazole sodium
129	Tetrabromofluorescein
130	Thiabendazole
131	Triethylamine
132	Trifluoperazine
133	Tylosin
134	Xylene Cyanol FF
135	β-Cyclodextrin
